# The training intensity distribution among well-trained and elite endurance athletes

**DOI:** 10.3389/fphys.2015.00295

**Published:** 2015-10-27

**Authors:** Thomas L. Stöggl, Billy Sperlich

**Affiliations:** ^1^Department of Sport Science and Kinesiology, University of SalzburgSalzburg, Austria; ^2^Integrative and Experimental Training Science, Department of Sport Science, University of WürzburgWürzburg, Germany

**Keywords:** high intensity training, high volume, low intensity, polarized training, prospective, pyramidal, retrospective, threshold training

## Abstract

Researchers have retrospectively analyzed the training intensity distribution (TID) of nationally and internationally competitive athletes in different endurance disciplines to determine the optimal volume and intensity for maximal adaptation. The majority of studies present a “pyramidal” TID with a high proportion of high volume, low intensity training (HVLIT). Some world-class athletes appear to adopt a so-called “polarized” TID (i.e., significant % of HVLIT and high-intensity training) during certain phases of the season. However, emerging prospective randomized controlled studies have demonstrated superior responses of variables related to endurance when applying a polarized TID in well-trained and recreational individuals when compared with a TID that emphasizes HVLIT or threshold training. The aims of the present review are to: (1) summarize the main responses of retrospective and prospective studies exploring TID; (2) provide a systematic overview on TIDs during preparation, pre-competition, and competition phases in different endurance disciplines and performance levels; (3) address whether one TID has demonstrated greater efficacy than another; and (4) highlight research gaps in an effort to direct future scientific studies.

## Introduction

The intensity and duration of work as well as recovery periods define overload and adaptations in athletes (Faulkner, 1968). While there appears to be consensus regarding the factors that limit endurance performance (Joyner, 1991; Coyle, 1995; Hawley and Stepto, 2001), agreement regarding the optimal volume and **training-intensity distribution** (TID) among elite athletes remains elusive. Achieving such consensus is important in order to maximize training adaptations and translate them into performance gains while avoiding overtraining.

KEY CONCEPT 1 **Training-intensity distribution**

The intensity of exercise and its distribution over time is one essential variable for prescribing the training stimulus. The training intensity is typically divided into zones on the basis of parameters such as heart rate, blood levels of lactate, gas exchange, power output or velocity, and/or perceived exertion.

Researchers have generally employed retrospective designs to analyze the TID of nationally or internationally competitive athletes in different endurance disciplines. In contrast, the number of prospective quasi-experimental or experimental studies investigating athlete responses to different TID's are small, with only limited studies examining well-trained or elite endurance athletes (Evertsen et al., 1997, 1999, 2001; Billat et al., 1999; Ingham et al., 2008, 2012; Yu et al., 2012; Stöggl and Sperlich, 2014). Articles reviewing the training intensity and duration of endurance athletes (Seiler and Tonnessen, 2009; Seiler, 2010) conclude that: (1) elite endurance athletes perform approximately 80% of their training at low intensity (< 2 mM blood lactate) with about 20% high-intensity work, (2) two **high-intensity training** (HIT) sessions per week suffice to induce adaptations for performance, and (3) the emphasis of HIT in highly trained athletes revealed equivocal results.

KEY CONCEPT 2 **High intensity training**

High-intensity or “zone-3” training (e.g., >4 mmol lactate/L blood, >90% maximal heart rate) involves mainly interval training, intermittent intervals, or burst-training (short, high-intensity sprints).

The percentage time spent in zones based on physiological benchmarks [derived from either heart rate (HR), gas exchange or blood lactate measurements], the session goal approach, and the session rating of perceived exertion (RPE) method have been applied to quantify the TID among endurance athletes (Seiler and Kjerland, 2006). Athletes may principally choose from one to four TIDs to induce endurance-related adaptations: (1) **high-volume, low-intensity exercise** (HVLIT), usually performed below the first ventilatory threshold (VT_1_) or at stable lactate concentrations of ≤ 2 mM and referred to as “zone 1” intensity; (2) **“threshold” training** (THR) performed at or near the lactate threshold (LT ~4 mM) or second ventilatory threshold (VT_2_) and designated as “zone 2” intensity; (3) HIT in “zone 3” (≥4 mM) (Seiler, 2010); or (4) a combination of the aforementioned concepts. Based on training analysis in elite rowers and cross-country skiers, a so called “**polarized**” TID has been proposed (Fiskerstrand and Seiler, 2004; Seiler and Kjerland, 2006). The polarized TID comprises significant % HVLIT time (i.e., “zone 1”) and HIT time (i.e., “zone 3”) compared to a low % THR time (“zone 2”). Some investigators have separated the TID into five zones (Tonnessen et al., 2014). In contrast, the traditional TID used in the bulk of previous investigations has been composed of a “**pyramidal**” structure (Holmberg, 1996), in which the majority of training time is spent in HVLIT (“zone 1”), and a decreasing proportion of training time in zones 2 and 3.

KEY CONCEPT 3 **High volume low intensity training**

Low-intensity training (e.g., below the first ventilatory threshold or at stable lactate concentrations < 2 mM) of longer duration, also referred to as long slow distance training or “zone-1” training.

KEY CONCEPT 4 **Threshold training**

Training performed mainly at an exercise intensity corresponding to the lactate threshold (e.g., 4 mM blood lactate) or second ventilatory threshold, involves primarily continuous or intervals of moderate-intensity exercise and is often defined as “zone-2” training.

KEY CONCEPT 5 **Polarized training**

The polarized training consists of significant proportions of both high- and low-intensity training and only a small proportion of threshold training. The distribution between low and high intensity training is often quantified as 80:20%, or 75–80% with low intensity, 5% threshold intensity, and 15–20% as high intensity training.

KEY CONCEPT 6 **Pyramidal training intensity distribution**

With the pyramidal distribution, most training is at low intensity, with decreasing proportions of threshold and high-intensity training.

Since nearly all studies dealing with TID were based on retrospective analysis, we recently employed a randomized controlled design to investigate which TID (HVLIT vs. THR vs. HIT vs. polarized) provided the greatest response on **key components of endurance performance** among well-trained athletes (Stöggl and Sperlich, 2014). We concluded that the polarized TID resulted in the greatest improvements in the majority of key endurance performance variables assessed, and THR or HVLIT did not lead to further improvements in performance. However, as numerous retrospective reports have shown conflicting results, the question regarding which TID represents the “best-practice” model for inducing performance gains—while avoiding overtraining—remains open to debate. Therefore, the aims of the present review were to: (1) summarize the main responses of different retrospective and prospective studies exploring TID; (2) provide a systematic overview of TIDs during preparation, pre-competition, and competition phases in different endurance disciplines and performance levels; (3) address whether one TID has demonstrated enhanced efficacy over another; and (4) highlight research gaps in an effort to direct future scientific studies.

KEY CONCEPT 7 **Key components of endurance performance**

In connection with many endurance sports five key parameters are utilized for comparison of performance: (1) peak oxygen uptake; (2) velocity or power output at the lactate threshold; (3) work economy; (4) peak running velocity or peak power output, and (5) time to exhaustion.

## Intensity distribution of endurance performance

### Retrospective studies

**Figure 1:**
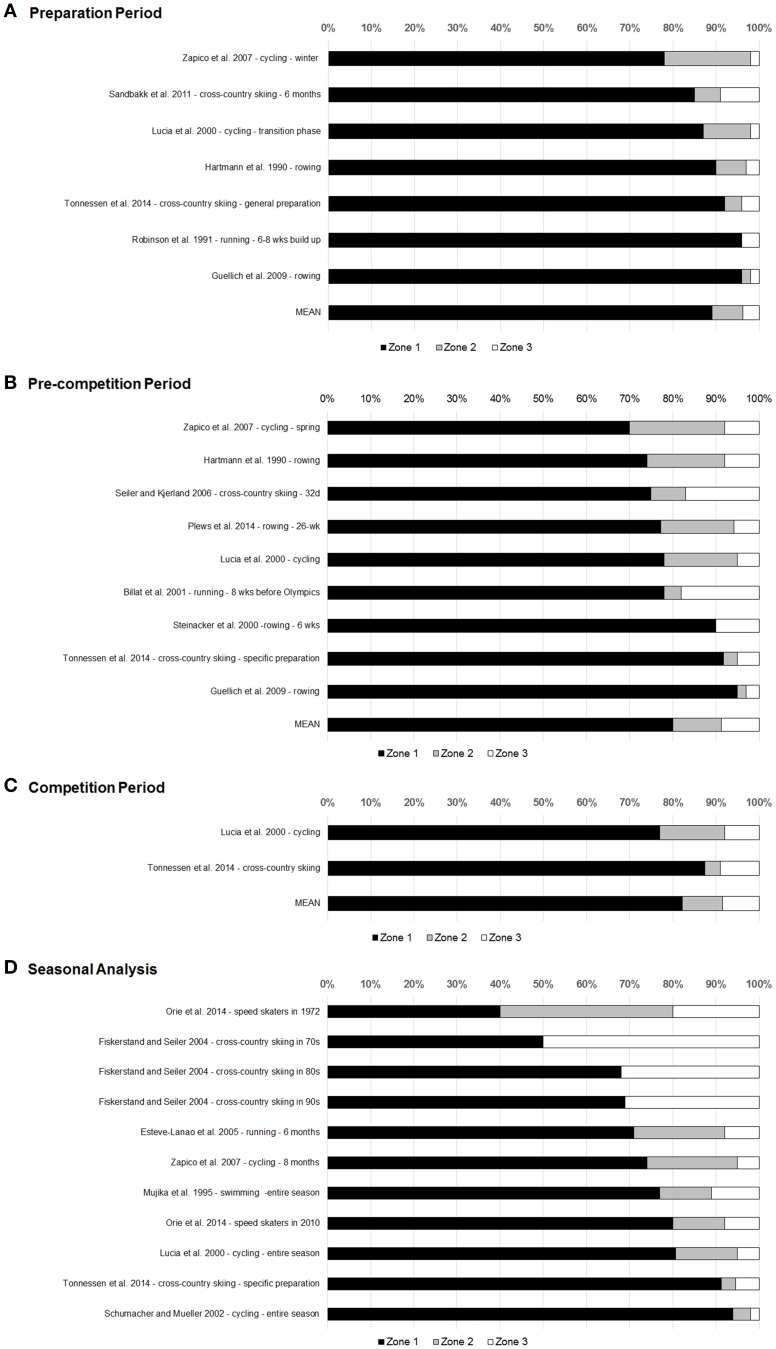
**The training-intensity distribution (i.e., percentage time spent in zone 1: < first ventilatory threshold or steady-state lactate at ~2 mM; zone 2: at or near lactate threshold (~4 mM) or second ventilatory threshold; zone 3: high-intensity training above lactate or second ventilatory threshold) in well-trained to elite endurance athletes in retrospective analyses during (A) preparation phase, (B) pre-competition phase, (C) competition phase, and (D) seasonal analysis**.

One major problem in TID investigations lies in the difficulty of involving elite athletes in a scientific experiment. Given their already high fitness levels, introducing certain novel training programs among elite performers may not result in performance enhancement and can even lead to overtraining symptoms. Therefore, the majority of studies dealing with TID in well-trained to elite endurance athletes are based on retrospective analyses of their training (Tables 1, 2, Figure 1).

**Table 1:** **Retrospective analysis of intensity-distribution during selected phases (e.g., preparation, pre-competition, competition phase) within a training year in well-trained to elite endurance athletes**.

**References**	**Sport**	**Subject characteristics**	**Research design**	**Intensity classification**	**Intensity zones**	**Intensity distribution**	
Robinson et al., 1991	Running	13 national-ranked male New Zealand, distances 1500 m to marathon; VO_2peak_ = 66.3 ml·min^−1^·kg^−1^ (61.3–70.1)	6–8 weeks (591 sessions) during the build-up phase, with most of the training as steady-state running. Racing and interval sessions were excluded from analysis (< 4% of all sessions)	HR during training converted to equivalent treadmill speeds and VO_2_	< LT (4 mM) >LT (4 mM)	96% 4%	
Steinacker et al., 1998	Rowing	German, Danish, Dutch and Norwegian elite junior rowers	Analysis of the preparation for World Championships	Based on blood lactate	1.5 mM ≥6.5 mM	75% 25%	
Steinacker et al., 2000	Rowing	German junior national team rowers of the coxed eight	Analysis of 6 weeks before the World Championships 1995	Based on blood lactate	< 4 mM ≥4 mM	90% 10%	
Billat et al., 2001	Running	11 Portuguese and 9 French national team runners. Top-class (marathon time < 2:12 h men; < 2:31 h women) vs. high-class (between 2:12 and 2:16 h). VO_2peak_: men 79.6 ml·min^−1^·kg^−1^, women 67.1 ml·min^−1^·kg^−1^	8 weeks before Olympic trials	Training classified according to duration and velocity	>marathon velocity = marathon velocity < marathon velocity	78% 4% 18%	
Billat et al., 2003	Running	Top-class male and female Kenyan long-distance runners (10 k: male 28:36, female 32:32, VO_2peak_: Male 78.4 ml·min^−1^·kg^−1^, Female 68.6 ml·min^−1^·kg^−1^)	Training logs of the final 8 weeks before the 10 k Kenyan Cross-Country Championships in 2002, separated into a high speed (HS) and low speed (LS) training group	Training classified according to duration and velocity	>90 min < vLT = vLT = vΔ50%-vLT to vVO_2max_ = vVO_2max_	HS 83.8% 6.9% 4.3% 0.0%	LS 84.2% 14.4% 1.4% 0%
Seiler and Kjerland, 2006	Cross-country skiing	Norwegian junior level cross-country skiers. VO_2max_ = 72.6 ml·min^−1^· kg^−1^. 10–12 h·wk^−1^ training	Retrospective analysis of a 32 d period during pre-competition phase (October–November)	HR time-in-zone; Session RPE;	Zone1: RPE ≤ 4, ≤ 2 mM, ≤ VT_1_ Zone2: RPE 4–7, 2–4 mM, VT_1_–VT_2_	~75% 5–10%	
				Blood lactate	Zone3: RPE ≥7, ≥4 mM, ≥VT_2_	15–20%	
Sandbakk et al., 2011	Cross-country skiing	Eight world class and eight national class Norwegian sprint skiers (VO_2max_: 70.6 vs. 65.8 ml·min^−1^·kg^−1^)	Retrospective analysis over 6 months during preparation phase (May–October)	Session goal approach	Zone 1: 1.5–2.5 mM, 60–81% HR_max_ Zone2, 2.5–4 mM, 82–87% HR_max_ Zone 3 > 4 mM, >88% HR_max_	Elite vs. National Class 84% 86% 7% 4.8% 8.7% 8.8%	
Plews et al., 2014	Rowing	9 heavy weight elite rowers (4 women, 5 men)	Retrospective analysis during the 26 weeks build-up to the 2012 Olympic Games	Training time in lactate zones	< LT_1_ LT_1_–LT_2_ >LT_2_	77.3% 16.9% 5.8%	

*HR, heart rate; LT, lactate threshold; vLT, velocity at lactate threshold; VT_1_, first ventilatory threshold; VT_2_, second ventilatory threshold; RPE, ratings of perceived exertion; HS, high speed; LS, low speed training group*.

**Table 2:** **Retrospective analysis of intensity-distribution (>6 months up to several years) in well-trained to elite endurance athletes**.

**References**	**Sport**	**Subject characteristics**	**Research design**	**Intensity classification**	**Intensity zones**	**Intensity distribution**			
Hartmann et al., 1990	Rowing	German elite rowers	Analysis between 1985 and 1988 during preparation and competition phase	Sessions within blood lactate zones	< 2 mM 2–4 mM 4–8 mM >8 mM		Prep. 86–94% 5–9% 1–4% 0–3%		Comp. 70–77% 15–22% 6% 2%
Mujika et al., 1995	Swimming	18 national and international class swimmers	Analysis over one season	Swimming speed based on blood lactate	2 mM 4 mM 6 mM 10 mM Maximal sprint			~77% ~12% ~6% ~4% ~1%	
Lucia et al., 2000	Cycling	13 professional cyclists VO_2max_ = 74 ml·min^−1^·kg^−1^	7 months comparing active rest, pre-competition and competition period	HR time-in-zone	< VT_1_ VT_1_ and VT_2_ >VT_2_		Rest 88% 11% 2%	Pre-Comp. 78% 17% 5%	Comp. 77% 15% 8%
Schumacher and Mueller, 2002	Team pursuit cycling	Seven cyclists of the German national pursuit team. VO_2peak_: 65–73 ml·min^−1^·kg^−1^. 4000 m individual time: 4:18.8–4:33.6	Analysis of the training year in preparation for the Olympic games in 2000 (March–September)	Based on blood lactate	< IaT = IaT >IaT			94% 4% 2%	
Fiskerstrand and Seiler, 2004	Rowing	28 Norwegian international medal winners. VO_2max_: 5.8–6.5 L·min^−1^	Analysis between 1970 and 2001. Test results of physiological testing (*n* = 28) and response to a detailed questionnaire via Email regarding training during their internationally competitive years (*n* = 21)	Described intensity zones	Long distance training (1–2.5 mM lactate) high intensity training			70 s: 50%: 50% 80 s: ~68%:32% 90 s: ~69%:31%	
Esteve-Lanao et al., 2005	Running	Regional and national class Spanish distance runners. VO_2max_ = 70 ml·min^−1^·kg^−1^	Retrospective analysis of a 6 month period with 4–5 h·wk^−1^ training (late August to mid-February)	HR time-in-zone	< VT_1_ VT_1_ and VT_2_ >VT_2_			71% 21% 8%	
Zapico et al., 2007	Cycling	14 elite U23 cyclists (VO_2max_ = 78 ml·min^−1^·kg^−1^)	Analysis over one season split into winter period (volume oriented) and spring period (intensity oriented)	HR time-in-zone	< VT_1_ VT_1_ and VT_2_ >VT_2_		Winter 78% 20% 2%		Spring 70% 22% 8%
Guellich et al., 2009	Rowing	36 young German male juniors from national rowing squad (31 international, 5 national junior finalists)	37 weeks divided into basic preparation, specific preparation, and early competition period. Comparison between national and international successful athletes 3 years later	HR control based on lactate	< 2 mM 2–4 mM >4 mM			95% 2% 3%	
Orie et al., 2014	Speed skaters	Successful Dutch Olympic speed skaters and long-track, middle and long-distance	Analysis over 4 Olympic seasons (1972–2010). Trainers, coaches, and athletes were interviewed and training diaries analyzed	Training time in lactate zones	< 2 mM 2–4 mM >4 mM		1972: 40% 40% 20%		2010: 80% 12% 12%
Tonnessen et al., 2014	Cross-country skiing	11 Norwegian elite cross-country skiers and biathletes (4 male: VO_2max_ = 85.1 ml·min^−1^·kg^−1^ and 7 female VO_2max_ = 72.9 ml·min^−1^·kg^−1^). All winners of at least one individual Olympic or World Championship senior gold medal from 1985 to 2011	Analysis using day-to-day recordings of training diaries in the year leading up to their most successful competition in their career	Time in training zone session goal	Zone 1 < 1.2 mM, 54–73% HR_max_ Zone2 1.3–2.0 mM, 74–83 HR_max_ Zone 3 2.1–3.6, 84–88 HR_max_ Zone 4 3.7–5.7 mM, 89–93 HR_max_ Zone 5 >5.8, >94% HR_max_	Prep. 87% 5.4% 3.6% 3% 1.2%	Pre-comp 88% 3.8% 3.1% 2.5% 2.5%	Comp 84% 3.6% 3.6% 4.5% 4.5%	Overall 86% 5.3% 3.3% 3.3% 2.1%

*HR, heart rate; IaT, individual anaerobic threshold; VT_1_, first ventilatory threshold; VT_2_, second ventilatory threshold; Prep, preparation phase; Comp, competition phase; Pre-Comp, pre-competition phase*.

#### Exercise-intensity distribution during the preparation period

From 1990 to 2014, the TID of elite, nationally ranked to world-class athletes who were training in their preparation phase were reported. These athletes competed in rowing (Hartmann et al., 1990; Guellich et al., 2009), running (Robinson et al., 1991), cycling (Lucia et al., 2000), and cross-country skiing (Sandbakk et al., 2011; Tonnessen et al., 2014). Findings indicate that elite endurance athletes spend a high percentage of their TID in a pyramid shape—that is, great portions of HVLIT with 84–95% in zone 1, 2–11% in zone 2, and 2–9% in zone 3 (Tables 1, 2, Figure 1A).

#### Exercise-intensity distribution before the competition phase

Depending on the competition calendar the TID during the pre-competition phase, may vary between endurance disciplines. The TID during pre-competition was analyzed in rowing (Hartmann et al., 1990; Steinacker et al., 2000; Guellich et al., 2009; Plews et al., 2014), running (Billat et al., 2001), cycling (Lucia et al., 2000), junior cross-country skiers (Seiler and Kjerland, 2006), and senior elite cross-country skiers and biathletes (Tonnessen et al., 2014) (Tables 1, 2, Figure 1B).

In elite rowers the TID during the pre-competition is inconclusive: in two studies the successful rowers decreased the proportion of HVLIT to 70–77% with increasing proportions of zone 2 up to 15–22%, and 5.8–6% in zone 3 (Hartmann et al., 1990; Plews et al., 2014). In contrast, two studies (Steinacker et al., 2000; Guellich et al., 2009) reported very high proportions of HVLIT (90–95%) during the pre-competition phase (i.e., 6 weeks before the 1995 World Championships).

In professional cyclists (Lucia et al., 2000) and top-class runners (Billat et al., 2001) engaged in pre-competition training, similar proportions of HVLIT were reported (78%). The distribution of zones 2 and 3 were however, polarized (4 and 18%) in the runners and pyramidal (17 and 5%) in the cyclists.

Comparable with the findings in several studies with rowers (Steinacker et al., 2000; Guellich et al., 2009), elite cross-country skiers and biathletes focus on HVLIT during the pre-competition phase (~91.8% zone 1–2 and 8.2% zone 3–5) (Tonnessen et al., 2014). However, the competitive junior cross-country skiers in the study of Seiler and Kjerland (2006) reported a polarized TID of 75, 5–10, and 15–20% in zones 1, 2, and 3, respectively, over a 32 days period during the pre-competition phase (End October, November).

Summarized, elite athletes in rowing (Hartmann et al., 1990; Steinacker et al., 2000; Guellich et al., 2009; Plews et al., 2014) and cycling (Lucia et al., 2000) reported pyramidal TID with HVLIT ranging from 78% in cycling up to 90–95% in some rowers, cross-country skiers, and biathletes. Billat et al. (2001) and Seiler and Kjerland (2006) reported a polarized TID with a greater proportion of zone 1 (75–78%) and zone 3 (15–20%) compared to zone 2 (4–10%).

#### Exercise-intensity distribution during the competition phase

Documentation of the TID during the competition period is rare since (a) technical equipment may not be applied during competition, (b) the TID largely depends on the amount and type of competitions (e.g., single races vs. stage races), and (c) the strategies for tapering for competitions vary widely across sports. Lucia et al. (1999) reported a pyramidal TID (70/23/7%) during the Tour de France based on the “HR time in zone” method over 22 competition days. The exercise intensity was particularly high during the time trials and high mountain stages. Also Lucia et al. (2000) reported that elite cyclists performed approximately 810 km·wk^−1^ (May) with a TID of 77/15/8%, while elite cross-country skiers and biathletes (Tonnessen et al., 2014) showed a higher proportion of HVLIT compared with THR and HIT (~87.5% zone 1–2 vs. ~12.5% zone 3–5) when compared with the cyclists (Figure 1C).

#### Exercise-intensity distribution based on seasonal analysis (up to 1 year) (Table 2, Figure 1d)

The TID covering a period of several months up to 1 year was reported in cycling (Lucia et al., 2000; Schumacher and Mueller, 2002; Zapico et al., 2007), swimming (Mujika et al., 1995), running (Esteve-Lanao et al., 2005), and cross-country skiing (Seiler and Kjerland, 2006; Tonnessen et al., 2014). Athletes from the different studies incorporated a high amount of HVLIT (70–94%), with variations in the amount of THR (4–22%) and HIT (2–11%), either as pyramidal or polarized TID.

In elite cyclists a trend from a nearly complete HVLIT (preparation period) toward pyramidal TID (pre-competition, competition period) can be observed. In a 7 month longitudinal study, professional cyclists (Lucia et al., 2000) increased both the training volume (267 vs. 713 vs. 810 km·wk^−1^, 15,000 total km) and intensity from active rest (88/11/2%) to pre-competition (78/17/5%) and competition phases (77/15/8%). Comparable findings were reported in U23 elite cyclists with a 78/20/2% TID during the winter (“volume mesocycle”) and 70/22/8% during the spring (“intensity mesocycle”) (Zapico et al., 2007). The recordings (29,000–35,000 km·yr^−1^) for the 4000 m team pursuit cycling world record in the year 2000 (excluding stage racing and track competitions), showed a main training focus on HVLIT with 94% < LT, 4% around LT, and 2% > LT (Schumacher and Mueller, 2002).

Comparable with the TID in the cycling studies during the pre-competition phase, regional- and national-class Spanish runners (4–5 h·wk^−1^) demonstrated a pyramidal TID of 71 (< VT_1_), 21 (VT_1_–VT_2_), and 8% (>VT_2_) (Esteve-Lanao et al., 2005) over a 6 month period. The TID of national and international-level swimmers revealed a pyramidal TID (although the athletes spent almost the same time in zone 2 and 3) over an entire season (77/12/11%) (Mujika et al., 1995). The Norwegian elite cross-country skiers and biathletes analyzed during the year leading to their most successful career competition (1985–2011) (Tonnessen et al., 2014) spent 91% of their training time in zones 1–2 and 9% in zones 3–5 or 77 vs. 23% when applying the session goal approach. The monthly frequency of HIT sessions and “zone 5” sessions increased from the general to the specific preparation period and remained unchanged within the competition period. From the end of the general preparation to the peaking phase, the amount of HVLIT decreased by 21%, and HIT—especially zone 5—increased by 40%. Therefore, the TID changed from an emphasis on HVLIT during preparation, toward a pyramidal TID during pre-competition, and a polarized TID during the competition phase.

#### Exercise-intensity distribution during long-term analysis (>1 year) (Table 2)

The TID across several decades was reported in rowers (Fiskerstrand and Seiler, 2004) and speed skaters (Orie et al., 2014). During the 1970s, the training volume of elite rowers was almost equally divided between HVLIT and HIT sessions (Fiskerstrand and Seiler, 2004). Then two major changes across time were identified: (1) training with low blood lactate (< 2 mM) increased from 30 to 50 h·month^−1^ and race pace and supra-maximal intensity training (8–14 mM) decreased from 23 to 7 h·month^−1^, and (2) total training volume increased from 924 (600–1020) h·yr^−1^ during the 1970s to 966 (840–1140) h·yr^−1^ in the 1980s, and to 1128 (1104–1200) h·yr^−1^ in the 1990s (~20% increase). Further increase in total training volume in the 1990s did not lead to further improvement in physical capacity.

Similar to the findings by Fiskerstrand and Seiler (2004), the TID of successful male Dutch Olympic speed skaters (Orie et al., 2014) in four Olympic seasons (1972–2010; assessed by interviewing the coaches and athletes) was based on THR in 1972 (40/40/20%), whereas the more recent TID was pyramidal (~80/~12/~8%) in 2010.

### Prospective studies investigating TID

#### Single case or quasi-experimental longitudinal studies without control groups (Table 3)

Based on three studies, we conclude that an increase in HVLIT at the expense of THR leads to performance enhancements, while the exaggerated increase in HIT at the expense of HVLIT might be applied with caution.

**Table 3:** **Non-experimental longitudinal or single case studies comparing different intensity-distribution in well-trained to elite endurance athletes**.

**References**	**Sport**	**Subject characteristics**	**Research design**	**Intensity classification**	**Intensity zones**	**Intensity distribution**	
Billat et al., 1999	Running	Eight endurance-trained male middle and long distance runners (1500 m to half-marathon). Training 6 sessions·wk^−1^	Non-experimental longitudinal study with 4 weeks using 4 HVLIT, 1 LT and 1 HIT session (5 × vVO_2max_ with 50% of tVO2max) followed by 4 weeks of 2 HVLIT, 1 LT, and 3 HIT sessions	Based on running speeds in %vVO_2max_	HVLIT (60–70% vVO_2max_ OBLA: 4 mM HIT: vVO_2max_	LOW 4 1 1	High 3 1 2
Ingham et al., 2012	Running	One international 1500 m runner (PB 3:38.9 min:s; VO_2max_:70.5–79.6 ml·min^−1^·kg^−1^)	Single case study over 2 years. In the first year the HVLIT was performed above the prescribed level and tempo training at an excessively high intensity. Second year HVLIT was increased from 20 to 55%, LT and HIT was reduced from 42 to 20% and 20 to 10%, while the highest intensity was increased from 7 to 10%	Based on respective speed expressed as & vVO_2max_	HVLIT: < 80% vVO_2max_ Tempo: 80–90% vVO_2max_ HIT: 90–100% vVO_2max_ Supramaximal: 100–130% vVO_2max_	Year1 ~20% ~44% ~20% ~16%	Year2 ~55% ~20% ~8% ~17%
Yu et al., 2012	Speed skaters	Nine Chinese top-level sprint skaters (500 m and 1000 m) all performing at World Cup and Olympic competitions during 2004–2006	Non-experimental longitudinal study comparing 1 year THR training vs. 1 year polarized training. Performances during national, World Cup and Olympic competitions and blood lactate after competitions were analyzed	HR time-in-zone based on lactate testings	Low: < 2 mM Moderate: 2–4 mM High: >4 mM	Year1 41% 51% 7%	Year2 86% 5% 10%

*HVLIT, high volume low intensity training; LT, lactate threshold; HIT, high intensity training; HR, heart rate; vVO_2max_, velocity at maximal oxygen uptake*.

Billat et al. (1999) examined whether one HIT session compared to three HIT sessions·wk^−1^ is sufficient to improve performance in four middle-distance runners. The implementation of four HVLIT, one HIT, and one THR session over 4 weeks resulted in improved running speed at maximal oxygen uptake (VO_2max_) and running economy. A further 4 weeks intensification, including two HVLIT, three HIT, and one THR session each week, showed no additional performance benefit, but increased subjective muscle stress, reduced sleep quality, and increased plasma epinephrine, all indicators of impending overtraining.

Altering TID from a THR-emphasis toward a more polarized or pyramidal TID showed improvements in competition performance and physiological capability. In the case study of a male international 1500 m runner (PB 3:38.9 min:s), HVLIT was performed within the first year above the coach's prescribed level and “tempo” training at an excessively high intensity. In the second year, HVLIT (< 80% VO_2max_) increased from 20 to 55% and intense training at 80–90% VO_2max_ and 90–100% VO_2max_ was reduced from approximately 42 to 20% and 20 to 10%, respectively. Furthermore, a concomitant increase in the proportion of HIT (100–130% vVO_2max_) from 7 to 10% was observed. This training modification coincided with improvements in physiological capability (increase in VO_2max_ from 72 to 79 ml·min^−1^·kg^−1^) and performance improvements (3:38.9 to 3:32.4 over 1500 m) (Ingham et al., 2012).

The 1 year adaptation of a THR emphasized (41/51/7%) vs. a polarized (86/5/10%) TID were evaluated in nine Chinese top-level sprint speed skaters (500 and 1000 m) and their performances at national competitions, World Cups, and the Olympics (Yu et al., 2012). The overall training durations and frequencies were similar across the two seasons, with THR constituting 50% of the training time (41/51/7%) and with POL 1 year later only 5% (86/5/10%). The increase in HVLIT, the reduction of THR from 50 to 5%, and the increase in HIT led to 2–4% improvements in the 500 m and 1000 m events.

#### Randomized controlled studies (Table 4)

The focus of the nine studies manipulating TID was mainly to compare the change in performance and/or adaptation to three different TID's including: THR-emphasized training, HVLIT-emphasized training, and polarized TID. In the majority of studies, recreational or sub-elite athletes were investigated. All experimental studies reported increased endurance performance, however, in most of the studies the polarized or HVLIT-emphasized TID resulted in the greatest responses of various endurance performance variables (Esteve-Lanao et al., 2007; Ingham et al., 2008; Neal et al., 2013; Munoz et al., 2014; Stöggl and Sperlich, 2014).

**Table 4:** **Randomized controlled trials (RCT) with different intensity-distribution in well-trained to elite endurance athletes**.

**References**	**Sport**	**Subject characteristics**	**Research design**	**Intensity classification**	**Intensity zones**	**Intensity distribution**			
Esteve-Lanao et al., 2007	Running	Twelve well-trained sub-elite Spanish runners (regional to national level, competition experience ≥5 years, VO_2max_ ~ 69 ml·min^−1^·kg^−1^, 5–6 h·wk^−1^ training	RCT with two groups performing the same amount of HIT but variations in the amount of HVLIT and THR while achieving equal training loads (TRIMP) over a 5 month period	HR time-in-zone and session goal approach	< VT_1_ VT_1_ to VT_2_ >VT_2_		LIT 80.5 (74) 11.8 (11) 8.3 (15)	THR 66.8 24.7 8.5	
Neal et al., 2013	Cycling	Twelve well-trained male cyclists from two local cycling clubs with consistent training >4 yr with 7–8 h·wk^−1^ training	Randomized cross-over design with POL distribution (80/0/20) and the THR distribution (57/43/0) over 6 weeks. Zone 3 training was performed indoor using 6 × 4 min intervals. Wash out period of 4 weeks. Muscle biopsies for mitochondrial enzyme activity, MCT 1&4 content and morning first-void urine was collected. Endurance performance assessed in a 40 km time trail, incremental exercise, PPO, and high intensity capacity (95% PPO to exhaustion)	HR time-in-zone	Zone 1: < LT Zone 2: PO 50% LT and LTP Zone 3: 5–10% greater PO at LTP		POL 80% 0% 20%	THR 57% 43% 0%	
Munoz et al., 2014	Running	30 recreational Spanish runners (mean competition experience ≥5.5 y; VO_2max_ POL: 61.0, THR 64.1 ml·min^−1^·kg^−1^)	RCT with a 10 weeks training program (5–6 sessions·wk^−1^) emphasizing POL (77/3/20%) or a moderately high –intensity program THR (46/35/19%) with equal volumes in zone 3 (2 sessions·wk^−1^ at ≥85% VO_2max_) and equal training load (TRIMP). 10 k running performance was analyzed	HR time-in-zone	< VT_1_ VT_1_ to VT_2_ >VT_2_		POL 72.9% 13.5% 13.6%	THR 46.8% 37.3% 15.8%	
Ingham et al., 2008	Rowing	18 experienced national standard British rowers	RCT, with randomization according to performance and selected physiological variables into two groups with identical training volume (~1140 km) with either 12 weeks HVLIT, or mixed intensity training (MIX)	Power output, 500 m split time and HR converted to equivalent % VO_2peak_	64–69% VO_2peak_ 70–75% VO_2peak_ 76–83% VO_2peak_ 84–93% VO_2peak_ 94–99% VO_2peak_ >100% VO_2peak_		HVLIT 53.8% 44.1% 0% 0.2% 0.9% 0.9%	MIX 49% 23% 0% 21.9% 3.2% 2.9%	
Evertsen et al., 1997, 1999, 2001	Cross-country skiing	10 well-trained Norwegian cross-country skiers competing at national and international junior level (11 men VO_2max_ 73.4 ml·min^−1^·kg^−1^, 9 women 58.3 ml·min^−1^·kg^−1^)	RCT with training for 5 months either mainly HVLIT or THR/HIT. Analysis of performance (20 min run test, VO_2max_ test, incremental test), biopsies for enzyme analysis, fiber typing, Na^+^-K^+^ pump and MCT1 and 4 content	Intensity control by HR and blood lactate	< 1.5 mM, 60–70% VO_2max_ HIT/THR: 3–4 mM, 80–90% VO_2max_ HIT		HVLIT 86% 14% 0%	THR 17% 83% 0%	
Seiler et al., 2013	Cycling	35 recreational cyclists (29 male, 6 female, VO_2max_ ~52 ml·min^−1^·kg^−1^ training volumes 6 h training·wk^−1^, 1.5 intervals·wk^−1^)	RCT trial with 4–6 training sessions·wk^−1^ over 7 weeks with intensity matched effort in a HVLIT group, and three interval training groups using intervals with differences in duration and intensity [2 times·wk^−1^ 4 × 4 min (94% HR_max_) or 4 × 8 min (90% HR_max_) or 4 × 16 min (88% HR_max_)]. Measures of VO_2peak_, peak power, power at 4 mM	Intensity distribution not presented. Duration of intervals in % total training time	Low intensity (≤ VT_1_) LT (88% HR_max_) 90–94% HR_max_	HVLIT 100% 0% 0%	4 × 4 91% 0% 9%	4 × 8 81% 0% 19%	4 × 16 72% 28% 0%
Stöggl and Sperlich, 2014	Running, cycling, cross-country skiing	48 well-trained endurance athletes from running, cycling, triathlon and cross-country skiing, VO_2peak_ = 62.6 ± 7.1 ml·min^−1^·kg^−1^	RCT comparing the effects of ~6 sessions·wk^−1^ for 9 weeks using HVLIT, THR, HIT, and POL	Session goal approach	LOW: < 2 mM LT: 3–5 mM HIGH: 90–95% HR_max_	HVLIT 83% 16% 1%	THR 46% 54% 0%	HIIT 3% 0% 57%	POL 68% 6% 36%

*HR, heart rate; HVLIT, high volume low intensity training; THR, lactate threshold training; LTP, lactate turnpoint; LT, lactate threshold; HIT, high intensity training; POL, polarized training; VT_1_, first ventilatory threshold; VT_2_, second ventilatory threshold; PPO, peak power output*.

Esteve-Lanao et al. (2007) were among the first to conduct experimental randomized controlled studies assessing the effects of 5 months increased or decreased HVLIT on endurance performance. Twelve sub-elite Spanish runners were randomly assigned to two separate groups performing equal amounts of HIT (8.4% of training > VT_2_). Although the two groups varied in the amount of HVLIT (group 1: 81%, group 2: 67%) vs. THR (group 1: 12%; group 2: 25%), they achieved equal total training loads (TRIMP scores). A polarized TID (74/11/15%) in the group with emphasized HVLIT was revealed with the session goal approach. The improvement in 10.4 km running was greater in the group emphasizing HVLIT (-157 vs. -122 s). If there is sufficient training time, it is advised to design an “easy-hard” rather than a “moderately high-hard” load distribution training approach.

In line with the Esteve-Lanao et al. (2007) study, experienced national standard British rowers performed either 12 weeks of HVLIT (98% ≤ LT) or mixed-intensity training (72% ≤ LT; 28% between LT and VO_2peak_) (Ingham et al., 2008). Whereas, both groups improved similarly in terms of their performance (VO_2peak_, 2000 m ergometer time trial), HVLIT improved performance at LT to a greater extent than in the mixed training group.

Neal et al. (2013) analyzed the molecular adaptation resulting from 6 weeks of polarized (80/0/20%) vs. THR (57/43/0%) TID in 12 male cyclists. In both groups, 40 km time trial performance, peak power output, power at LT, monocarboxylate-transporter 4 and high-intensity exercise capacity all increased; however, improvements were greater with polarized TID concerning peak power output (8 vs. 3%), power at LT (9 vs. 2%), and high-intensity capacity (85 vs. 37%), despite greater total training volume in THR (458 vs. 381 min·wk^−1^).

Munoz et al. (2014) manipulated the TID in 30 recreational runners randomly assigned to a 10 weeks training program (5–6 sessions·wk^−1^) emphasizing polarized training (77/3/20%) or THR (46/35/19%) with equal volume in zone 3 (i.e., 2 weekly sessions at ≥85% VO_2max_) and equal in training load (TRIMP). Both groups increased their 10 km performance by 5.0 vs. 3.5% for polarized vs. THR TID. In a sub-analysis of selected athletes with TIDs emphasizing either zone 1 in the polarized group or zone 2 in the THR group, the polarized TID showed greater improvements (+7.0%) compared with THR (+1.6%).

Stöggl and Sperlich (2014) explored the response of HVLIT (83/16/1%) vs. THR (46/54/0%) vs. HIT (43/0/57%) vs. polarized TID (68/6/26%) on key components of endurance performance in 48 well-trained runners, cyclists, triathletes and cross-country skiers. While all four groups increased time to exhaustion, the polarized TID increased VO_2peak_ (+11.7%), time to exhaustion (+17.4%), and peak performance (+5.1%) to the greatest extent. Performance at 4 mM increased after polarized TID (+8.1%) and HIT (+5.6%), with no change in the other groups. HIT resulted in decreased body mass (-3 kg) and increased VO_2peak_ (+4.8%). Exclusive emphasis of THR or HVLIT did not lead to further improvements in endurance performance in well-trained athletes.

Evertsen et al. (1997, 1999, 2001) randomly assigned 20 well-trained Norwegian cross-country skiers for 5 months to HVLIT vs. a HIT/THR emphasized TID. The HVLIT group spent 86% at an intensity < 1.5 mM (7 sessions·wk^−1^ with an increase from 10 to 16 h·wk^−1^) and 2–3 sessions·wk^−1^ HIT (14%), while the HIT/THR group demonstrated 83% HIT/THR at 3–4 mM (12 h·wk^−1^) and 17% HVLIT as recovery. Despite a 60% increase in training volume in the HVLIT group and approximately four times more training at intensity close to LT in the HIT/THR group, physiological and performance changes were modest in both groups. In contrast to other studies (Ingham et al., 2008; Stöggl and Sperlich, 2014), greater improvements in performance (e.g., running speed at LT and performance in a 20 min run) were found in the HIT/THR group compared with HVLIT.

Seiler et al. (2013) analyzed the performance adaptations of different types of high intensity interval training. Thirty-five recreational cyclists were randomized to four training groups with equivalent training the two previous months (~6 h·wk^−1^, ~1.5 session·wk^−1^). The HVLIT group trained 4–6 sessions·wk^−1^, and the three HIT groups trained two sessions·wk^−1^ with either 4 × 4 min (94% HR_max_), 4 × 8 min (90% HR_max_), or 4 × 16 min (88% HR_max_) plus 2–3 sessions·wk^−1^ HVLIT. The 4 × 8 min interval group induced greater average gains in VO_2peak_, power at VO_2peak_, and power at 4 mM. Subjects without interval training experience before the intervention tended to achieve greater average improvements in VO_2peak_, peak power output, and power at 4 mM compared with subjects reporting 1–1.5 HIT sessions·wk^−1^. All three interval training groups tended to improve in physiological capacity after the training period, while the HVLIT group remained relatively unchanged (with the exception of a significant increase in power at 4 mM), despite similar or slightly higher total training volumes (8.5 h·wk^−1^ vs. 5.7–7.6 h·wk^−1^).

## Methodological considerations

Although the authors are aware that the investigations summarized in this review vary regarding the endurance disciplines, athletic level, duration of observation, time of season (preparation, pre-competition, or competition phase) and TID methodology, the present data show no uniform TID pattern among the different endurance disciplines (Figure 1).

The methodology incorporated in the retrospective analysis to compare the TID between disciplines might produce discrepancies in numbers. The intensity of endurance exercise is frequently defined as percentage of HR_max_ or VO_2max_ or blood lactate concentration. The percentages of for example, VO_2max_ have been shown to lead to inhomogeneous metabolic strain as indicated by the large variability of blood lactate responses (Scharhag-Rosenberger et al., 2010), and therefore may lead to imprecise assignment to the intensity zones during prolonged exercise.

Elite endurance athletes also implement strength training and speed training in their conditioning training. Since these training forms incorporate short (< 30 s), very intense, anaerobic bouts, the continuous measurement of for example HR (due to inertia of the cardio-respiratory system at the onset of intense exercise) may preclude exact quantification of the intensity zone for this part of training. In this context, other methods, such as the “session RPE method” or the “session goal approach” (Seiler and Kjerland, 2006) might be applied. For other candidate biomarkers to quantify training load and understand fatigue in athletes we refer to Halson (2014).

## Intensity distribution among enurance athletes

Most retrospective studies report a pyramidal TID, with extensive HVLIT (>70%), less time in zone 2, and very little time spent in zone 3, independent of the time of season. Three studies on polarized TID involving cross-country skiers during different phases of the season (Seiler and Kjerland, 2006; Sandbakk et al., 2011; Tonnessen et al., 2014) and one on marathon runners (Billat et al., 2001) were found.

Athletes favor HVLIT, since when the training volume is high, low intensity training (< 2 mM or ~55–85% HR_max_) is more tolerable (Hartmann et al., 1990). Despite athletes' preference for low intensity training the majority of coaches favored higher intensity [2.5–4 mM (THR)], mainly for theoretical reasons (e.g., Fritsch, 1985, 1986; Nolte, 1986). Since the amount of HVLIT has been linked to improved race performance (Hagerman and Staron, 1982; Steinacker, 1993; Esteve-Lanao et al., 2005; Seiler and Kjerland, 2006), the necessity of HVLIT in achieving physiological adaptations for gains in performance has been pointed out in longitudinal observations and experimental designs (Esteve-Lanao et al., 2005, 2007; Zapico et al., 2007; Ingham et al., 2008). However, when the amount of HVLIT by elite athletes is doubled, no further improvement in performance is evident (Costill et al., 1991), and the athletes mood may be negatively affected (Raglin, 1993). Therefore, for elite endurance athletes with high amounts of HVLIT, the ability to distribute the training intensity optimally may be paramount to both success and counteracting non-functional overreaching (Fiskerstrand and Seiler, 2004).

The longitudinal retrospective observations (Fiskerstrand and Seiler, 2004; Orie et al., 2014) and quasi-experimental designs (Ingham et al., 2012; Yu et al., 2012), revealed a THR-emphasized TID in recent decades. Overdoing THR by >20% through reducing HVLIT may exert a negative impact on the autonomic nervous system (Chwalbinska-Moneta et al., 1998; Esteve-Lanao et al., 2007), with no further adaptation (Esteve-Lanao et al., 2007; Guellich and Seiler, 2010; Ingham et al., 2012; Yu et al., 2012; Neal et al., 2013; Stöggl and Sperlich, 2014). In fact, THR training places greater demands on carbohydrate fueling, leading to restricted training time due to limited glycogen storing (Beneke et al., 2011). However, THR may be more applicable for untrained and/or recreational individuals (Kindermann et al., 1979; Yoshida et al., 1982; Denis et al., 1984; Keith et al., 1992; Takeshima et al., 1993; Londeree, 1997; Gaskill et al., 2001). In some disciplines however, displaying a pyramidal TID, THR was thought to be a fundamental part of the training program (7–22%) of elite endurance athletes or in distinct phases of the season (Hartmann et al., 1990; Lucia et al., 2000; Esteve-Lanao et al., 2005; Zapico et al., 2007; Sandbakk et al., 2011; Plews et al., 2014).

The various responses to HIT have been investigated extensively, showing rapid adaptions of various tissues and performance indexes (Lindsay et al., 1996; Weston et al., 1997; Stepto et al., 1999; Laursen, 2010) involving aerobic and anaerobic energy demands (Laursen and Jenkins, 2002; Laursen, 2010; Sperlich et al., 2011). The molecular events (Laursen, 2010), fueling strategies (Burke, 2010), hydration (Maughan and Shirreffs, 2010), psychological skills (Birrer and Morgan, 2010), and overtraining prevention (Kellmann, 2010) in connection with HIT have been previously described in detail.

Runners who have prioritized HIT instead of THR into their HVLIT- training have been reported to perform better (Billat et al., 2003), a result which was corroborated in a prospective study (Stöggl and Sperlich, 2014). Approximately two HIT sessions·wk^−1^ have been proposed to stimulate performance adaptations without inducing chronic stress (Seiler, 2010). While it was shown that an increase from one to three HIT sessions per week was not accompanied with further performance benefits, such an increase did result in greater subjective muscle stress, plasma epinephrine, and reduced sleep quality, all indicators of impending overtraining (Billat et al., 1999).

Although there are numerous time-efficient adaptations and health benefits associated with HIT, there is evidence that individuals will need to feel physically capable and adequately motivated to perform and maintain high intensity exercise (Hardcastle et al., 2014). Additionally, condensed HIT over a longer period (9 weeks) may lead to a loss in body mass in well-trained athletes (Stöggl and Sperlich, 2014) which may also negatively impact health.

Training adaptation is subject to high inter-individual response (Bouchard et al., 1986), and disciplines with high eccentric forces, high force impacts (e.g., running), and chronic muscle damage will not necessarily exhibit similar TID when compared to disciplines with lower eccentric impact (e.g., swimming, cycling, rowing) because recovery and low-intensity exercise might be less prominent. From this point of view the same TID will in all likelihood induce different adaptations among individuals, even if they behave and exercise in an “elite” manner.

Although the number of retrospective studies reporting a HVLIT or pyramidal based TID is substantial compared to polarized TID in well-trained to elite endurance athletes (Tables 1–4, Figure 1), the findings from various prospective studies (≤ 5 months training intervention), suggest that a polarized TID results in superior training and performance responses compared to HVLIT and THR in some endurance disciplines and certain phases of the season. Since, variation of the training stimuli is a critical aspect of effective training (Kiely, 2010, 2012), switching from a long-term unidirectional training stressor (e.g., HVLIT) to another training stressor that provides a substantial increase in the amount of HIT (e.g., polarized TID) may prove fruitful. The optimal type of periodization model however, remains open for debate (Issurin, 2010; Kiely, 2010, 2012). For instance, it is noteworthy that the effects of an inverse pyramidal or inverse polarized TID (applying a high amount of HIT with lower portion of HVLIT—e.g., 20:80), or exclusive HIT for a longer period (>9 weeks) has not been analyzed. Based on the experience of our previous investigation (Stöggl and Sperlich, 2014), researchers—especially those conducting prospective studies—will have to face the question which TID (in combination with different periodization models) is superior in athletic and health seeking populations.

Technical advancements integrating different internal (e.g., HR, oxygenation via near-infrared spectroscopy, sleep analysis, breathing pattern, HR variability, hand-held analysis of saliva and blood, questionnaires, etc.) and external (power output, GPS-based distance and speed, accelero- and inclinometer, etc.) sensor technologies that enable biological monitoring at a distance (Chan et al., 2012) will further enhance the estimation of individual athletes' optimal TID in a timely manner. Yet, the question remains: which data are the best foundation for quantifying TID?

In summary, most retrospective studies on well-trained to elite endurance athletes report a pyramidal TID, with a large proportion of HVLIT. Polarized TID has been proven to be an effective strategy for some elite athletes during certain phases of the season. However, experimental studies lasting 6 weeks to 5 months demonstrate superior responses to polarized TID, especially when compared with TID that emphasizes THR or HVLIT. As pointed out, the combination of HVLIT with HIT may improve endurance performance with potentially less autonomic and hormonal stress and boredom. The reasons for the non-uniform TID among endurance disciplines may arise from differences in methodology in retrospective analyses and/or high inter-individual variation in the training response. Furthermore, the long-term effects of different forms of TID (e.g., inverse pyramidal or inverse polarized or exclusive HIT) with different patterns of periodization on well-trained to elite endurance athletes, have yet to be characterized. Consequently, an “optimal” TID cannot be identified, and future prospective randomized investigations conducted over extended time-periods will have to be designed to address this question.

### Conflict of interest statement

The authors declare that the research was conducted in the absence of any commercial or financial relationships that could be construed as a potential conflict of interest.
